# Continuous Spinal Anesthesia for Obstetric Anesthesia and Analgesia

**DOI:** 10.3389/fmed.2017.00133

**Published:** 2017-08-15

**Authors:** Ivan Veličković, Borislava Pujic, Charles W. Baysinger, Curtis L. Baysinger

**Affiliations:** ^1^Department of Anesthesiology, SUNY Downstate Medical Center, Brooklyn, NY, United States; ^2^Klinika za Ginekologiju I Akuserstvo, Klinickog Centra Vojvodine, Novi Sad, Serbia; ^3^Department of Anesthesiology, University of Kentucky Medical Center, Lexington, KY, United States; ^4^Division of Obstetric Anesthesia, Department of Anesthesiology, Vanderbilt University School of Medicine, Nashville, TN, United States

**Keywords:** obstetric anesthesia, labor analgesia, intrathecal catheters, neuraxial blockade, post-dural puncture headache, spinal catheters

## Abstract

The widespread use of continuous spinal anesthesia (CSA) in obstetrics has been slow because of the high risk for post-dural puncture headache (PDPH) associated with epidural needles and catheters. New advances in equipment and technique have not significantly overcome this disadvantage. However, CSA offers an alternative to epidural anesthesia in morbidly obese women, women with severe cardiac disease, and patients with prior spinal surgery. It should be strongly considered in parturients who receive an accidental dural puncture with a large bore needle, on the basis of recent work suggesting significant reduction in PDPH when intrathecal catheters are used. Small doses of drug can be administered and extension of labor analgesia for emergency cesarean delivery may occur more rapidly compared to continuous epidural techniques.

## Introduction

Continuous spinal anesthesia (CSA) is an anesthetic technique that offers several clinical advantages for anesthesia and analgesia in obstetric patients. The level of sensory blockade can be titrated to the desired dermatomal level with great precision with intrathecal (IT) catheters, allowing better control of the hemodynamic consequences of sympathetic blockade associated with spinal anesthesia compared to epidural or single shot spinal techniques (1). Better control of maternal hemodynamics maybe advantageous in patients with cardiac disease in whom administration of lower doses of local anesthetics is advantageous. In addition, cerebrospinal fluid (CSF) aspiration allows a visual endpoint for catheter position and allows for a rapid confirmation of catheter placement. Similar to epidural catheters, an IT catheter can be re-injected during longer surgical procedures (2); however, only a fraction of the local anesthetic dose is required for effective analgesia and anesthesia compared to epidural techniques. It also has a more rapid onset and delivers a denser sensory block compared to epidural anesthesia (2). It becomes an option in those patients with prior spinal surgery where other neuraxial techniques may not be effective and if unintentional dural puncture during an epidural placement occurs, particularly in the morbidly obese patient in whom the risk for dural puncture headache may be lower.

The PubMed database was searched using terms “Intrathecal catheters” and “Spinal catheters” and all articles, from 1964 to January 2017, were examined by the authors. Publications that contained data in obstetric patients were identified for further review, as well as review articles and meta-analyses. A search of the references of review articles and meta-analysis were also preformed to identify other publications that might have been missed in the PubMed database search.

## Historical Perspectives

Although CSA was first described approximately 100 years ago in surgical patients (3), it was not until 1944 that its use for labor analgesia and cesarean delivery was reported (4, 5). Nearly 45 years of improvements in catheter technology were required before small-bore catheters were developed that could be placed through small-bore needles, which would reduce the rate of post-dural puncture headache (PDPH) to an acceptable level. In December 1987, Hurley and Lambert described 28–32 gauge microcatheters that could be inserted through 26 G spinal needles (6). CSA might have become a widely used technique for labor analgesia with an acceptably low risk of PDPH, but Rigler et al. reported four cases of cauda equina syndrome associated with their use in 1991 when 5% lidocaine was administered (7). By the end of 1991, an additional seven cases had been reported to the United States Food and Drug Administration (FDA) (2). As a result, the FDA issued a safety alert on May 29, 1992: “We are concerned that the use of small-bore catheters in CSA is increasing, and thus that the potential for new cases of cauda equina syndrome associated with this technique may be increasing. Because of these safety concerns, we are advising against the use of any small-bore catheter for CSA of any local anesthetic agent (8).” Although the FDA recognized that the pooling of intrathecally administered drug, which exposed nerve roots to high concentrations of local anesthetic, was the most probable cause of the reported neurotoxicity (1), small-bore catheters were subsequently removed from the commercial market (2).

Since 1992, spinal microcatheters using a through the needle system have been used in the U.S. only in clinical trials. In 2008, Arkoosh et al. found that the quality of analgesia for labor with a 28 G IT catheter was as good as that of an epidural after insertion through a 22 gauge needle (9). The study also found a 9% incidence of PDPH (epidural group 4%) with 5.3% of patients requiring an epidural blood patch (epidural group 2%). These findings occurred with an incidence that approached statistical significance and no persistent neurologic deficit was noted in either group (9). At present, no IT microcatheters using a catheter through the needle system are commercially sold in the USA, but are available in Europe (Spinocath^®^ BBraun Melsungen AG, Germany).

In Europe and, more recently in the USA, experience with catheter over the needle designs for CSA has been reported. Alonso et al. used this system for 92 elective cesarean deliveries using a 24-gauge catheter placed over a 26 gauge SprotteTM needle (10). A 14% failure rate was described, far higher than the 2% rate reported by Arkoosh, but they used low amounts of isobaric bupivacaine that might explain the high rates of failure (11). A high rate of PDPH (28%) was also reported, but the rate of epidural blood patch placement was low (6% overall). Much better success was reported by Tao et al. using the same system as Alonso, with no reported failures, no reported neurological deficits, and an incidence of PDPH similar to that reported by Arkoosh et al. (8.8%) (12).

Most recently, Wiley Spinal (Wiley Spinal^®^, Epimed Int., Johnstown, NY, USA) has begun marketing an FDA approved, IT catheter system in the USA using the over the needle system. Whether this design will be adopted for widespread clinical use is unclear. In an observational series of 113 laboring women, Dresner and Pinder failed to successfully place an IT catheter in 11% using this newer system (13), a rate much higher than that reported for either combined spinal–epidural or epidural catheter placement. However, successful use of an IT catheter for spinal anesthesia for cesarean delivery was reported in 94% of women who required an operative delivery and with a low rate (2.6%) of PDPH. Others have reported significant paresthesias and a much higher rate of PDPH (14). At this point, there is insufficient experience with catheter systems designed for IT anesthesia and analgesia to define reliable rates of successful placement, catheter failure, transient complications during placement or PDPH, and prolonged complications of neurological deficit.

### Indications for IT Catheter Use

Despite the potential availability of the abovementioned IT catheter systems, the only catheters that are in widespread use by a majority of U.S. anesthesiologists are 19 and 20 gauge epidural catheters inserted through 17 or 18-gauge Tuohy needles designed for epidural placement (1). In addition, some epidural kit manufacturers offer smaller gauge catheters (typically 24 gauge) and smaller bore needles (most often 20 gauge) intended for pediatric epidural placement, which might reduce the headache rate with the use of smaller diameter equipment. The high rates of PDPH (approximately 60% of whom will receive an epidural blood patch) (15) limits larger bore IT catheters to non-routine use. However, there are some clinical scenarios where CSA is attractive (Table 1).

**Table 1:** Clinical indications for CSA.

Clinical indication	Comments
Maternal cardiac disease	– Slow titration of sensory level with small incremental boluses allows patient and operator adjustment to sympathetic blockade
Morbid obesity	– Possible decreased rate of catheter failures compared to epidural placement – Probable modest decrease in PDPH rate compared to non-obese parturients
Prior spinal surgery	– High rate of epidural block failure (up to 40%) makes CSA an attractive alternative
Accidental dural puncture	– Avoids risk of further dural puncture during difficult epidural placement – PDPH rate may be reduced with continuous IT catheters

*CSA, continuous spinal anesthesia; IT, intrathecal; PDPH, post-dural puncture headache*.

In the patient with severe cardiac disease where hemodynamic stability is very important (for example, the patient with valvular stenotic disease or severe myocardial dysfunction), case series (13) and numerous case reports (16, 17) show that CSA can afford excellent anesthesia with minimal hemodynamic perturbation. Spinal analgesia for labor can be accomplished by use of catheter injection of opioids alone (1). Anesthesia for surgical delivery can be accomplished by very slow titration of local anesthetic allowing gradual patient compensation to the physiologic changes of sympathetic blockade.

In patients with prior spinal surgery (Harrington rod placement for scoliosis, spinal fusion, and prior trauma), epidural placement of a catheter for labor analgesia may be impossible, and the changes in the epidural space will often limit epidural solution spread and effectiveness. Difficult epidural placement and a failure rate of approximately 40% had been reported in such patients (18). CSA offers a satisfactory alternative in the patient in whom neuraxial blockade is desired for labor or prolonged cesarean delivery.

Morbidly obese patients often present significant challenges for neuraxial catheter placement and have much higher rates of epidural catheter failure and emergency abdominal delivery (19). In addition, the rate of unintentional dural puncture is higher (19). Continuous spinal anesthesia offers an attractive alternative, providing more rapid titration of anesthesia for cesarean delivery with greater assurance of block success (CSF aspiration is an objective positive test for correct catheter placement). In addition, morbid obesity may convey protection against PDPH compared to non-obese parturients if an IT catheter is used. A number of retrospective case controlled and cohort studies suggest modest reductions of up to 20% in the overall headache rate (20). Whether the rate of epidural blood patching is reduced is controversial with some studies showing no effect (20) and others showing a 50% reduction in the rate of epidural blood patching (21).

In cases of accidental dural puncture during epidural catheter placement, placing the catheter into the IT space is a reasonable alternative to resiting the epidural needle. A recent survey found that anesthetists at 59% of maternity units in the United Kingdom prefer to use an epidural catheter as a spinal catheter in the case of accidental dural puncture. The two most common reasons for IT catheterization were to avoid further dural puncture (76%) and to allow immediate analgesia for labor (75%) (22).

### Clinical Management

No prospective trials examining optimal medication administration for CSA have been reported. However, our review provides information adequate for recommendations on the dosing of CSA for labor and cesarean delivery and we present such as per our local institutional protocols (Table 2). For labor analgesia, bolus doses 10% of epidural dosing and epidural continuous infusion have been suggested by some (23), but many authors have found that larger doses are required. A 2 ml/h infusion of a commonly used mixture of local anesthetic and fentanyl for epidural infusion (0.1% ropivacaine + fentanyl 2 µg/ml or 0.0625% bupivacaine + fentanyl 2 µg/ml) is a recommended starting regimen (12, 24). Since standard epidural catheters can have up to 1 ml of dead space, a priming volume equal to the catheter volume will be required during the initial dose. Breakthrough pain can be treated with either provider bolus doses of this solution or with the use of a patient controlled pump. If desired, 1 ml of saline flush may be used for clearing of the catheter with each bolus dose if intermittent dosing by provider is chosen. If patient controlled pumps are available, boluses of 2 ml of the same solution every 15 min, with a maximum of three boluses per hour, have the theoretical advantage of reducing the risk for infection by avoiding multiple manipulations of the IT catheter that may increase the risk for catheter contamination (25). A relatively wide range of dosing may be encountered.

**Table 2:** Suggested management of CSA.

Technique	Regimens at Authors’ Institutions
CSA for labor analgesia^a^	Continuous infusion: – 0.1% ropivacaine or 0.0625–0.1% bupivacaine with 2 µg/ml of fentanyl at 2 ml/h – Provider/patient controlled top ups, 1–2 ml every 15 min up to three doses per hour Intermittent bolus: – 2 ml of 0.1% ropivacaine or 0.0625–0.1% bupivacaine with 15 µg of fentanyl every 2 h
CSA for cesarean delivery	– Initial dose of 5–7.5 mg of 0.5% isobaric bupivacaine or 0.75% hyperbaric bupivacaine – Titrate dose to desired level with 2.5 mg incremental blouses every 3–5 min

*CSA, continuous spinal anesthesia*.

*^a^Standard epidural catheters (with Flat Filter) have up to 1 ml of dead space; priming of catheter to fill a catheter’s dead space is required on first bolus dose*.

A continuous spinal catheter placed for labor analgesia can also be extended for cesarean delivery (Table 2). An initial dose of 5 mg of 0.5% isobaric bupivacaine with fentanyl and titration doses in 2.5 mg increments, or similar amounts of 0.75% hyperbaric bupivacaine is recommended (Table 2). A priming volume equal to the catheter volume will be required with an initial dose to insure drug delivery.

A recent study has shown that the ED 50 and ED 95 of spinal hyperbaric bupivacaine are 6.7 and 11 mg, respectively (26). If the goal is to start a cesarean section rapidly, doses closer to the ED 95 are appropriate. If the block becomes inadequate during the procedure, it can easily be extended with intermittent bolus dosing. Because CSA techniques allow careful titration of dose with significant reduction of the local anesthetic amount, decreases in rates of maternal hypotension, vasopressor requirements, maternal nausea, time to discharge from the PACU, and improved maternal satisfaction might occur when compared to one-shot spinal techniques (27).

### Complications Associated with CSA

#### CSA and PDPH

Intrathecal catheter use may reduce the risk for PDPH over that associated with dural puncture with a large bore needle. While the mechanism (or mechanisms) of PDPH are only partially understood, most investigators accept that persistent loss of CSF is a significant cause (15). Although the rate of CSF leakage has not been correlated to headache severity, leaving a catheter *in situ* for 24 h might decrease CSF leakage by inducing an inflammatory response around the catheter site that would help seal the arachnoid–dural rent (28). Sjoberg et al. demonstrated the presence of a fibrous capsule around epidural/spinal catheter at its entrance into spinal space in patients with long-term IT catheters (29). Ayad et al. reported a 3% epidural blood patch rate, and the authors speculated that an inflammatory response due to prolonged catheter insertion might have led to such a favorable result (28). However, other retrospective reports (24) and one prospective trial (20) have failed to duplicate these results. An IT catheter also allows saline injection to reduce PDH rate and severity. Charsley and Abram noted that 10 ml of saline injected into the IT space following accidental dural puncture during epidural placement reduced the headache rate, but best results occurred if an epidural catheter was placed intrathecally and 10 ml of saline injected through the catheter just prior to removal at the end of anesthesia (30). Kuczkowski and Benumof reported a case series of seven patients where CSF in the glass syringe was injected back into the subarachnoid space through the epidural needle, and a small amount of preservative-free saline (3–5 ml) was injected into the subarachnoid space through the IT catheter (31). PDPH occurred in one of the seven patients in this small case series. Both of these reports emphasize the importance of maintaining CSF volume in reducing the severity and rate of PDPH.

Based on current meta-analyses, prolonged IT catheter insertion, after the accidental dural puncture, has a potential to completely change the way that we try to prevent PDPH. The 2008 systematic review by Van de Velde et al. showed that prolonged IT catheter placement reduced the risk for PDPH by only a small amount (51 vs. 66%), but significantly reduced the need for epidural blood patch (59 vs. 33%) (32). In 2010, Apfel et al. reported that the majority of interventions for the prevention of PDPH showed little efficacy, but the immediate placement of an IT catheter or the use of a prophylactic epidural blood patch before epidural catheter removal should be considered. No firm recommendations on pharmacologic agents to avoid PDPH after accidental dural puncture could be made (33). Heesen et al. reported in 2013 that IT catheter placement significantly reduced the need for an EBP, although a significant reduction in the incidence of PDPH was not seen (34). Verstraete et al. retrospectively reviewed anesthetic records from 29,749 neuraxial blocks (35). These authors reported that IT catheter placement significantly reduced the incidence of PDPH to 42% compared with 62% in patients who had epidural catheter placed at another level after ADP. These four systematic reviews report modest reductions in the rate of PDPH, and more significant reductions in epidural blood patch administration, thus implying that patients with an IT catheter have milder headaches. Although the only prospective trial to examine whether IT catheters reduce the incidence of PDPH failed to show a reduction in PDH and epidural blood patch rates (36), that study was stopped early and Heesen commented that “a type-2 error cannot be excluded as a cause of the lack of effect of IT catheterization on PDPH frequency” (34).

Conservative treatment of PDPH can be attempted in mild-to-moderate cases. Although caffeine has been used for almost 70 years, a 2007 systematic review by Halker et al. (37) showed no benefit from its use. However, a more recent systematic review reported in 2011 reached an opposite conclusion (38). In this report, Basurto et al. stated “caffeine has shown effectiveness for treating PDPH, in decreasing the proportion of participants with PDPH persistence, and in those requiring supplementary interventions, when compared with placebo” (38). The use of IT morphine and oral frovatriptan has shown initial positive results in small trials (38, 39). In addition, one well-done, randomized, prospective, controlled study, gabapentin significantly reduced pain, nausea, and vomiting compared to an ergotamine/caffeine combination in patients with PDPH (40). A combination of acetaminophen/butalbital/caffeine (FioricetTM) is also commonly used by the authors for the treatment of mild-to-moderate PDPH.

A PDPH prevention/treatment protocol might be employed in the way that is similar to that reported by Kuczkowski (41). Following accidental dural puncture, an epidural catheter is placed intrathecally. The catheter is then used as an IT catheter and removed 24 h later. Prior to the removal of the catheter, 10 ml of normal saline is administered through the IT catheter. The patient is also advised to take P.O. pain medications as per her obstetrician’s preference. In the case of persistent headache, a blood patch is performed. Strict sterile technique (i.e., hand washing, face mask, and sterile gloves) is mandatory during the placement of the IT catheter and during its manipulation to reduce the risk for infection. The use of an in-line bacterial filter and careful labeling are required to alert all caregivers of the location of the catheter.

#### Other Complications

Rates of other complications associated with the IT use of epidural catheters placed are low. In 2016, Cohn et al. reported on the complications associated with 761 short-term IT macrocatheters in obstetric patients over 12 years period (42). There were no serious complications, including meningitis, epidural or spinal abscess, hematoma, arachnoiditis, or cauda equina syndrome associated with IT epidural catheters.

## Conclusion

The CSA technique has significant drawbacks that limit its routine use in obstetric anesthesia. The rate of PDPH, when newer spinal catheters designed for CSA are used, is considerably higher than that reported with epidural catheters and CSE techniques (43). The intentional use of CSA should be considered in special circumstances where single shot subarachnoid block, CSE, or epidural block may be undesirable, such as prior spinal surgery, morbid obesity, or severe cardiac disease. Catheters designed for epidural use are those most widely available to U.S. anesthesiologists and have rates of PDPH higher than that reported with newer equipment. No study directly compares their IT vs. epidural effectiveness for labor analgesia or for cesarean delivery anesthesia and is an area for further research. Recent systematic analyses show modest reductions in rates of PDPH and larger reductions in epidural blood patches when epidural catheters are used intrathecally, following ADP. This suggests that IT catheters should be placed preferentially over resiting of an epidural catheter in those settings where IT catheters can be used safely. Routine use of CSA may occur only after the development of equipment and techniques that reduce the rate of PDPH and difficulties in placement over that currently available.

## Author Contributions

IV, BP, CWB, and CLB declare that (1) they made substantial contributions to the conception or design of the work and the acquisition, analysis, or interpretation of data for the work; (2) they drafted the work and revised it; (3) they approve its publication; and (4) they agree to be held accountable for all aspects of the work in ensuring that questions related to the accuracy or integrity of any part of the work are appropriately investigated and resolved.

## Conflict of Interest Statement

The authors declare that the research was conducted in the absence of any commercial or financial relationships that could be construed as a potential conflict of interest.
